# Real-Time Virtual Support as an Emergency Department Strategy for Rural, Remote, and Indigenous Communities in British Columbia: Descriptive Case Study

**DOI:** 10.2196/45451

**Published:** 2023-12-22

**Authors:** Helen Novak Lauscher, Brydon Blacklaws, Erika Pritchard, Elsie Jiaxi Wang, Kurtis Stewart, Jeff Beselt, Kendall Ho, John Pawlovich

**Affiliations:** 1 Department of Emergency Medicine University of British Columbia Vancouver, BC Canada; 2 Rural Coordination Centre of British Columbia Vancouver, BC Canada; 3 Faculty of Medicine University of British Columbia Vancouver, BC Canada

**Keywords:** telemedicine, access, rural, emergency medicine, interprofessional collaboration

## Abstract

**Background:**

British Columbia has over 200 rural, remote, and Indigenous communities that have limited health care resources due to physician isolation, sparsity in clinical resources, the lack of collegial support, and provider burnout. Real-time virtual support (RTVS) peer-to-peer pathways provide support to patients and providers. Amid the COVID-19 pandemic exacerbating existing health care disparities and equitable access to timely care, RTVS presents a portable and additional opportunity to be deployed in a hospital or patient home setting in rural communities. We highlight the story of the Rural Urgent Doctor in-aid (RUDi) pathway within RTVS that successfully supported the Dawson Creek District Hospital (DCDH) emergency department (ED) in 2021.

**Objective:**

This study aims to describe the rapid implementation process and identify facilitators and barriers to successful implementation.

**Methods:**

This case study is grounded in the Quadruple Aim and Social Accountability frameworks for health systems learning. The entire study period was approximately 6 months. After 1 week of implementation, we interviewed RUDi physicians, DCDH staff, health authority leadership, and RTVS staff to gather their experiences. Content analysis was used to identify themes that emerged from the interviews.

**Results:**

RUDi physicians covered 39 overnight shifts and were the most responsible providers (MRPs) for 245 patients who presented to the DCDH ED. A total of 17 interviews with key informants revealed important themes related to leadership and relationships as facilitators of the coverage’s success, the experience of remote physician support, providing a “safety net,” finding new ways of interprofessional collaboration, and the need for extensive IT support throughout. Quality improvement findings identified barriers and demonstrated tangible recommendations for how this model of support can be improved in future cases.

**Conclusions:**

By acting as the MRP during overnight ED shifts, RUDi prevented the closure of the DCDH ED and the diversion of patients to another rural hospital. Rapid codevelopment and implementation of digital health solutions can be leveraged with existing partnerships and mutual trust between RTVS and rural EDs to ease the pressures of a physician shortage, particularly during COVID-19. By establishing new and modified clinical workflows, RTVS provides a safety net for rural patients and providers challenged by burnout. This case study provides learnings to be implemented to serve future rural, remote, and Indigenous communities in crisis.

## Introduction

Rural health care providers serve over 200 rural, remote, and Indigenous communities across a wide range of geographically diverse areas in British Columbia [1]. As a collective community of practitioners, these providers encounter unique clinical challenges related to their geographic disposition [1-4], such as working in isolation, restricted clinical resources, and the lack of collegial support, which contribute to provider burnout [5,6]. The COVID-19 pandemic exacerbated health care disparities in these communities, which further magnified the inequity of access to primary and acute care. During COVID-19, digital health (ie, using information and communication technologies to provide health care and peer-to-peer support for health professionals) changed dramatically with the introduction of more portable and sophisticated equipment such as digital tablets or smartphones, more common videoconferencing software that is easy to use, and increasing options to connect to the internet in higher speed including from homes [7]. These developments have liberated digital health to be deployed much more nimbly anywhere in the hospital or even from patients’ homes to meet rural needs.

In April 2020, a provincial network of digital health pathways, collectively known as real-time virtual support (RTVS), was established in British Columbia to improve health equity in rural, remote, and Indigenous communities. Multiple organizations collaborated to create a foundation of trusted partnerships with the shared goal of improving health care for rural, remote, and Indigenous community members and health care providers. Founding organizations include the British Columbia Ministry of Health, Rural Coordination Centre of British Columbia (supported by the Joint Standing Committee on Rural Issues), First Nations Health Authority, Provincial Health Services Authority, Providence Health Care, British Columbia Emergency Medicine Network, and University of British Columbia Department of Emergency Medicine. RTVS was rolled out rapidly and implemented flexibly to adapt to the changing COVID-19 pandemic and problems faced by communities.

The RTVS network connects health care providers and patients to RTVS virtual physicians via Zoom (Zoom Technologies, Inc) or telephone [8-11]. RTVS has two main types of service: (1) peer-to-peer support for urgent and nonurgent situations, including case consultations, second opinions, ongoing patient management, transport coordination, point-of-care ultrasound, and simulation-based training and (2) direct-to-patient care that offers citizens and Indigenous community members direct remote access to health professionals in British Columbia.

This paper will focus on an RTVS peer-to-peer pathway that serves rural, remote, and Indigenous communities in British Columbia—the Rural Urgent Doctor in-aid (RUDi) pathway—created for rural health care providers to receive decision support for urgent cases [8,9]. The RUDi service is available to all rural health care providers in British Columbia on a 24-hour, 7-day per week basis. RUDi virtual physicians are selected for their strong knowledge and understanding of the rural context and, in most cases, have experience working in remote locations. At the time this paper was written, there were 30 RUDi physicians on the roster, with 2 available at any given time. During a typical 12-hour RUDi shift, the virtual physician is available on-demand by Zoom and telephone. Incoming calls are answered directly without a “front-door” operator. This distinguishes RUDi from other services in British Columbia, such as the Rapid Access to Consultative Expertise Line [12]. From April 2020 to January 2022, RUDi provided 16,104 hours of coverage and 2838 hours of clinical time to 75 rural communities across British Columbia, with rising demand for this service by rural health providers over time. For example, in its first month of service (April 2020), RUDi supported 27 calls from rural providers, whereas by March 2021, RUDi supported 286 calls. In January 2022, RUDi was handling over 450 calls per month.

Specifically, this paper documents a situation in which the RUDi service was deployed to provide most responsible provider (MRP) support remotely to a rural emergency department (ED) in a time of crisis to avert the diversion of patients to a hospital in another community. This shifted RUDi from being a decision support service to providing clinical emergency telemedicine. The RUDi service was originally designed and implemented to provide decision support for providers practicing in rural and remote areas. When the staffing crisis occurred in Dawson Creek, RUDi stepped in to provide coverage to the Dawson Creek District Hospital (DCDH) ED remotely and act as the MRP remotely. It was the first, and at that time, the only community that was supported by RUDi in this way.

Emergency telemedicine has a longstanding history of serving rural and remote populations. In 2012, a pilot of emergency telemedicine in western Australia supported rural EDs to manage patients locally and reduce the need for transfer to larger centers [13,14]. Closer to home in Canada, Nova Scotia implemented collaborative emergency centers to address gaps in rural care [15]. This telemedicine service provided 24/7 emergency care access across the province and reduced unplanned ED closures. Improvements in work-life balance were experienced by local rural physicians. A recent systematic review of telemedicine in rural and remote EDs highlighted some promising results [16]. In over half of the papers reviewed, there were improvements in time-sensitive clinical effectiveness including increased timeliness of care. Around one-third of the studies reviewed showed significant improvement in the appropriateness of transfer.

The remainder of the paper documents the case through the perspectives of those involved including RUDi physicians, Dawson Creek ED staff, health authority leaders and administrative staff, and RTVS support personnel. It tells the story of the collaborative development of the strategy through to its implementation and reflections on the experience shortly afterward.

## Methods

### Overview

We approached the evaluation of this intervention as a descriptive case study [17] situated within the broader RTVS evaluation. The overall RTVS evaluation approach is grounded in the Quadruple Aim [18] and Social Accountability [9,19] frameworks, with emphasis on provider experience and equity, respectively. The evaluation team, guided by a partnership-based advisory committee, agreed that the Quadruple Aim, including population health; patient and provider experience; and cost factors underpinned with social accountability values of relevance, quality, and equity, were aligned with the equity-informed approach of RTVS. The larger RTVS evaluation collects data across end user communities. Dawson Creek was a pathfinding situation in that other communities have needed, requested, and used the service since. Notably, RUDi now steps in as the MRP for remote nursing stations as part of its standard operating procedure. The Dawson Creek coverage was the first of its kind, and this rapid evaluation and reflection was useful in preparing for and adaptively implementing it in subsequent communities.

Semistructured key informant interviews [20] were used together with a review of relevant documents [21]. The case was used as a “Plan-Do-Study-Act” cycle [22] to share learnings and generate insights among participants and RTVS stakeholders for the improvement of the service, and documentation of learnings for future use in other communities. Because of the rapid nature of the evaluation of this emergent use of RTVS and the need to quickly learn from it, we quickly undertook reflection interviews with key participants to enable a rapid cycle of reporting and feedback to inform future implementation. For this rapid quality improvement evaluation, patient and family caregiver interviews were out of scope. An element of snowball sampling was incorporated in that we asked participants if there was anyone else involved in the Dawson Creek coverage that we should speak with.

### Setting

Dawson Creek is 1 of 13 Rural Practice Subsidiary Agreement communities located in northeastern British Columbia with a population of 11,574 [23]. The DCDH ED serves 25,000 patient encounters per year from the community and Peace River Regional District and receives referrals from at least 2 other smaller communities. The high patient volume is served with 31 acute care beds; a 15-bed regional psychiatric ward; and, in May 2021, a team of fewer than 25 physicians, including 7 general specialists and 7 family physicians who support ED coverage on a rotational basis [24]. DCDH was dealing with an ongoing shortage of physician coverage for its ED.

### Sampling and Participants

Sampling for document analysis included key emails, meeting minutes, and project documentation spanning approximately 1 month prior to implementation (to capture planning) and 2 months after implementation to capture relevant information. For key informant interviews, purposive sampling was used to identify participants from among RUDi physicians, DCDH staff, Northern Health Authority (NHA) leadership, and RTVS technology and administrative leaders or staff involved. Individuals were contacted by email and invited to participate in a 30- to 60-minute interview via videoconference or Zoom or telephone if they preferred. A total of 17 interviews were conducted with RUDi physicians (n=6), DCDH providers (n=4), NHA leadership (n=4), RTVS technology and administrative personnel (n=2), and an administrative staff member (n=1) in a partnering organization (local division of family practice) who was heavily involved in preparation for implementation.

### Data Collection and Analysis

Semistructured key informant interviews were conducted to examine the experiences and perspectives of the providers, leaders, and administrators involved in the development and implementation of this ED coverage strategy. The interview guide was codeveloped with RTVS and RUDi leads who would be involved in the setup and implementation, along with site leads and the evaluation lead or teams. It was designed to collect practical feedback and also document the impact on the practice setting and health system. The interview guide (Textbox 1) included questions related to process, successes, challenges, outcomes, facilitators, and suggestions for improvement.

**Interview guide.**

- Please take a moment to introduce yourself, your role, and how you were involved.
- What challenges were faced by the community? From your perspective, what was the situation?
- How did this effort get initiated? What happened? Who was involved? What was the chain of events in setting it up and what steps were taken to respond?
- To what factors do you attribute the ability to mobilize this way?
- What did you foresee as challenges going in? What challenges were experienced? Is there anything you would have done differently?
- What were some of the successes experienced? Are there notable cases or situations or experiences you would like to elaborate on?
- What would have happened without this response? Overall for the community? For the site? For individuals?
- What is something you took away from the experience? Is there anything you would have done differently?
- What were some of the notable learnings that came out of this experience from your perspective? Is there anything that could have been done differently? How could what you’ve learned help future planning?
- What would need to happen for it to be replicable or sustainable? Note barriers and facilitators
- What is your vision for how this type of response could fit into the system? What downstream benefits do you foresee?
- Is there anything else you’d like to add that you didn’t get a chance to talk about?
- Who else should we talk to? (note names or contact and roles)

Interviews were conducted by 4 RTVS evaluation team members who regularly participated in RTVS and RUDi team meetings. Interviews were all conducted using videoconference via Zoom and were recorded; they occurred within 2 months of implementation of this strategy. Interviews were transcribed by the RTVS evaluation team members. Transcripts were analyzed using the constant comparative method [25] to identify themes. Documents such as meeting minutes, emails, health care use records, and other process documentation were reviewed and included in the thematic analysis. The evaluation team members discussed ongoing analysis. An initial summary of findings was shared with participants for verification in the context of Plan-Do-Study-Act discussions. A timeline of key dates is included in Textbox 2. We used the Standards for Reporting Qualitative Research [26] to guide our reporting activities.

**Timeline of key dates.**

April 7, 2021: Earliest date of documents to be considered for analysis

May 7, 2021: First date of initial most responsible provider coverage or implementation

May 13, 2021: Last date of initial most responsible provider coverage or implementation

May 18, 2021: Interview data collection begins

June 29, 2021: Preliminary analysis complete and shared

July 1, 2021: Last interview collected

July 15, 2021: Additional analysis including final interview

Sept 19, 2021: Study period ends, integrative analysis, and report

### Ethical Considerations

Research ethics board approval was not obtained as this work is part of a program evaluation and quality improvement initiative and does not require ethics approval as per the Tri-Council Policy Statement: Ethical Conduct for Research Involving Humans. An ethics waiver can be supplied upon request [27]. In conducting the interviews, we followed the ethics standards for informed consent and for maintaining confidentiality and security of the data. We reviewed confidentiality, data use, and storage procedures with participants prior to interviews and asked participants for their verbal consent to conduct and record the interviews. No compensation was provided for participation in the interviews.

## Results

### Overview

The first 2 subsections summarize the results from the document analysis, and the remainder of the subsections present findings specific to the interviews.

### Cocreating the Virtual ED Coverage Strategy

Given the ongoing physician shortage, DCDH physicians were experiencing burnout and could not take on additional ED shifts. The NHA was preparing for the DCDH ED to temporarily close overnight and go on diversion, with incoming emergent patients redirected to the nearest ED in Fort St. John, about 80 km (45-60 minutes) away by a vehicle. It should be noted that both hospitals have similar challenges with physician recruitment and retention.

Less than 1 week before the anticipated start of the ED diversion, the Northeast Health Service Delivery Area’s Medical Director reached out to RTVS and RUDi to discuss alternatives to diversion and how RUDi could potentially help. Prior to this time, RUDi had provided occasional support to DCDH ED physicians via RTVS, so a relationship already existed to build upon. Within 48 hours of this initial discussion, a plan was conceived in which RUDi physicians would be on-call and the MRP for DCDH ED from 10:00 PM to 8:00 AM. Local physicians would resume coverage of the ED and their offices from 8:00 AM to 10:00 PM. Over the next 72 hours, this overnight coverage plan was shared and discussed with DCDH, NHA, RTVS, and RUDi teams, and implementation details were confirmed.

NHA, DCDH, and RTVS IT personnel worked together to rapidly grant the required administrative and medical privileges to 25 RUDi physicians, thereby allowing them to remotely access DCDH diagnostic charts, share medical records via fax, and have permission to practice at DCDH. RUDi physicians and DCDH ED nursing staff met prior to roll-out to coordinate how combined remote and in-person care would work. They codeveloped a triage plan where RUDi physicians would be the MRP for all Canadian Triage and Acuity Scale (CTAS) level 2-5 patients [28], with CTAS level 2-3 patients being seen by RUDi, while level 4-5 patients were monitored by nurses and held overnight unless their condition worsened. CTAS level 1 patients were seen in person by the on-call DCDH ED physician.

### Implementing the Virtual ED Coverage Strategy

Starting May 7, 2021, RUDi physicians provided overnight coverage of the DCDH ED for 7 consecutive nights. There were 3 to 4 nursing staff on the DCDH floor for each overnight shift. They were able to videoconference on demand with the on-call RUDi physician via a tablet attached to a rolling stand. This configuration could be moved from bed to bed in the ED and maneuvered around a patient for visual examinations, with the patient able to see and interact with the physician. An examination of 7 nights of coverage will be presented through interviews with individuals involved in planning and implementing the coverage strategy, namely, health leaders and administrators, support staff, community providers, and RUDi.

From May 7 through September 19, 2021, RUDi provided 39 shifts of overnight coverage for the DCDH ED. RUDi took the first call for a total of 245 patients in the ED, 236 (96.3%) of which were unscheduled visits. Overall, 41.2% (n=101) of patients were CTAS level 3, a total of 31% (n=76) were CTAS level 4, and 18.8% (n=46) were CTAS level 2. The majority (n=218, 89%) of unscheduled patients were discharged home, with 9 leaving against medical advice and 8 transferred to another facility RUDi directly supported a range of typical patient cases seen in the DCDH ED such as patients presenting with symptoms of stroke, mental health issues, and severe abdominal pain.

As noted, the following subsections present findings specific to the interviews. The themes identified are discussed in turn beginning with enabling factors, namely foundational relationships; supportive leadership; extensive IT support; and interprofessional collaboration, including perspectives of RUDi physicians and DCDH nurses. Finally, challenges experienced, along with solutions identified are presented.

### Foundational Relationships

When the crisis hit, partners relied upon long-established mutual trust. Strong relationships among RTVS, DCDH, and NHA leads allowed for efficient decision-making so that a solution could be established quickly—within 48 hours before the anticipated closure of the ED. Mutual trust between the health authority and RTVS and RUDi leadership and commitment to building good relationships between RUDi doctors and DCDH nurses were essential to rapidly develop and implement this solution.

Furthermore, existing professional working relationships between the DCDH staff and RUDi physicians accelerated the development of an interprofessional clinical workflow for this new configuration. Furthermore, the DCDH ED staff had prior experience in telemedicine initiatives through the implementation of Critical Outreach and Diagnostic Intervention in 2018 [29,30]. The implementation of iPads for Critical Outreach and Diagnostic Intervention situated the hospital with a receptive impression of RTVS. The awareness that this iPad was already located in the hospital and only required an update to set up the RUDi program was key to improving the community acceptance of the RUDi as a means to support the ED: “Having a pre-existing relationship was crucial [to establishing the coverage].”

### Supportive Leadership

The leadership shown by DCDH and NHA staff motivated DCDH ED nurses to work with this new strategy and prepared them to reach out to RUDi physicians as they usually would. NHA’s direct support enabled the efficient setup of RUDi physicians to practice in their health authority. With adequate credentials and an accelerated support process, RUDi physicians then were able to be set up within 1-2 hours of their Dawson Creek coverage shifts. Interorganizational collaboration at the leadership level was efficient and practical and allowed for swift decision-making. The shared goal of preventing diversion was described by a senior health administrator as an “opportunity to be proactive and get in front of a crisis*.*” In the development and implementation of this ED coverage strategy, those involved across roles and organizations, from senior leadership to staff, viewed all involved as a team. As one RUDi physician noted: “This is ground-breaking work...result of a massive amount of team effort that overcame a lot of unknowns to do something that was really important” [RUDi virtual physician interview 1]. Furthermore, a willingness to take risks in order to meet an urgent need and achieve a worthy goal provided impetus to the team. An RTVS IT leader explained: “There was no hesitation...absolutely we can do this…this is what this team is for.”

### Extensive IT Support

Extensive support was provided by IT personnel from DCDH and RUDi, including precoverage setup (eg, updating iPad software at DCDH ED and provisioning the required credentials for RUDi physicians), spending 1-2 hours before each shift assisting RUDi physicians (eg, accessing the NHA web-based portals and faxing records), and troubleshooting issues throughout the shifts. It was a prodigious and complex undertaking, not without risk, that required many planning meetings and creative problem-solving. NHA directly facilitated the physician credentialing process so RUDi physicians had access to the different patient charting systems.

Resourcefulness, collaborative problem-solving, and “going above and beyond” for a shared goal were identified as characteristics of the IT team members. A RUDi physician described the centrality of IT support to the success of the coverage:

From getting access to Northern Health, setting-up two-factor authentication, accessing Power Chart and Faxing patient information, walkthroughs of unfamiliar hospital programs, the IT support was key to ensure patient-centred and quality care.

RUDi virtual physician interview 6

### Interprofessional Digital Collaboration

Relationships between the Dawson Creek nurses and RUDi were cultivated through meetings to develop a workflow that took place in the evenings before the start of the Dawson Creek coverage. This familiarity helped the team work together remotely. Nurses were initially hesitant to trust the doctors from other communities but were open to connecting due to (1) lowering access barriers in advance of this event through the implemented iPads and (2) strong leadership at the site and ED level. It was difficult to “get the nurses confident in actually using the app...and be confident it would be a good experience” noted one of the community providers [community provider interview 1]. Similarly, for the RUDi physicians, there “were a lot of unknowns” in terms of local dynamics. Although most RUDi physicians had awareness and some experience practicing in rural settings, most RUDi physicians had not yet worked with this particular community. Nurses not only needed to communicate patient information but also had to “educate” RUDi physicians:

If you [have] never been in a rural town, you probably will not have an idea of how it works. What is available and what is not available in the hospital and community in terms of resources and services when delivering patient care.

Community provider interview 4

Nurses expressed that they felt increased pressure due to the RUDi physician’s remote presence versus a physician being in the room. For example, 1 nurse was uncomfortable with providing a neurological examination that a RUDi doctor suggested, as it was out of the scope of practice for the nurse. This required a shift in usual collaborative practice: “the nurse had to understand that they have help, but it’s not a physical person” [RUDi virtual provider interview 3]. While some nurses beforehand were not confident it would be a good experience, they found the experience to be positive overall, as one of the community providers stated:

It was something new, one of the nights did not go well...a guy came in with rapid edema...it was an uncomfortable situation but the RUDi doctor was awesome.

Community provider interview 1

RUDi physicians were generally positive about assuming the MRP role at DCDH ED. On their regular RUDi service shifts, they were given the option of trading the remote ED coverage shifts if they wished to opt out. A small number of RUDi physicians expressed discomfort with this new type of coverage, which placed greater medicolegal responsibility on them, leading to some physicians choosing not to take part.

DCDH medical staff noted that the digital coverage highlighted ongoing issues of physician recruitment and retention and the need for long-term solutions. They also expressed that new methods of interprofessional collaboration were implemented in order to support nurses. Even though participating remotely, RUDi physicians were able to advocate with compassion and urgency for nurses to have in-person physician support for high-acuity patients when needed. For example, several cases were passed from RUDi physicians to DCDH specialists and physicians, who attended patients in the hospital in the morning. RUDi physicians also highlighted the difficulty of digitally assessing and dispositioning patients; thus, many lower-acuity patients were asked to return to the ED in the morning for in-person care. However, this is a similar process to what is implemented at DCDH ED, with the DCDH physicians not finding this an issue.

### Challenges and Solutions

Participants identified challenges or areas of improvement. Risks associated with the challenges were identified. Risks experienced during the implementation were identified as “real” and those risks that were anticipated in ongoing implementation as “potential.” As the interviews were part of ongoing improvement efforts, planning for addressing anticipated risks was as important as describing how challenges experienced were addressed. Therefore, some of the potential risks were prevented during implementation by applying a solution that was collaboratively developed. Also identified were solutions that were many of which were tried out during implementation and documented for future use. Key challenges identified were technology integration into the nurses’ clinical workflow, shift handover, ongoing IT troubleshooting, and lack of local knowledge from RUDi. Table 1 provides a summary of challenges and solutions described by participants in the interviews.

**Table 1:** Summary of challenges, risks, and solutions identified by participants for service improvement.

Challenge and risks		Solutions	Illustrative quotes
**Technology integration into nurse’s workflow**			
	Potential: delays connecting to RUDi^a^ and patient assessment	Interim: setting up easy-to-use technology; patient, collegial support provided by RUDi physicians; real-time IT support Future: additional preparation and simulations for nurses before shifts to increase familiarity	“[It was difficult to] get the nurses confident in actually using [Zoom] and be confident it would be a good experience” [community provider interview 1].
**RUDi to DCDH^b^ shift handover**			
	Potential: loss of clinical information	Interim: faxing RUDi notes to DCDH, which are printed and added to patient charts Future: NHA^c^ transitioning to electronic records; integrating record systems used by NHA and RUDi	“From getting access to Northern Health, setting-up two-factor authentication, accessing Power Chart and faxing patient information…. was key to ensure patient-centred care” [RUDi virtual physician interview 6].
**Lack of local area knowledge from RUDi**			
	Real: 1 patient was mistakenly diverted to a nearby ED^d^, thereby delaying care Real: additional burden on nurses to explain setting and resources	Future: additional training and education for RUDi physicians before shifts	“If you have never been in a rural town, you probably have no idea how it works” [Community provider interview 4].
**Expectation mismatch**			
	Potential: additional pressure on nurses to perform procedures that RUDi physicians cannot Real: a nurse declined to perform an “out-of-scope” neurology examination requested by the RUDi physician	Interim: patients are held overnight until they can be seen by a DCDH physician Future: additional training with nurses and RUDi physicians to clarify roles and scope of practice	“The nurse had to understand that they have help, but it is not a physical person” [RUDi virtual provider interview 3].

^a^RUDi: Rural Urgent Doctor in-aid.

^b^DCDH: Dawson Creek District Hospital.

^c^NHA: Northern Health Authority.

^d^ED: emergency department.

Learnings and insights such as those described here were discussed in team meetings in the weeks after the virtual ED coverage was implemented. Solutions identified were defined as either “interim,” to deal with during the coverage, or “future,” to denote ongoing quality improvement. The novel service model offered highly original training for both RUDi physicians and DCDH nurses: “This put everyone in place and set the stage for an amazing learning opportunity from which to build and improve on collaboration moving forward.” The documentation of the experience contributed to a tool kit cocreated by partners for use in future RTVS or RUDi implementations in other community EDs.

## Discussion

### Principal Findings

Our findings provide insights from a variety of perspectives into developing and implementing a remote emergency care strategy tailored to the needs of a local setting in crisis. The findings underscore the importance of foundational relationships, supportive leadership, and collaborative planning and cocreation of this strategy. The resourcefulness and dedication of IT support to facilitate and troubleshoot solutions were key facilitators. Interprofessional and intermodal (ie, via digital means) collaboration was necessitated in the implementation of the strategy. The willingness to work together and come together to reflect on challenges to inform future implementation was documented through engagement in this rapid evaluation process. These insights offer practical learnings transferable to similar situations, as exemplified by the cocreation of the toolkit and ongoing documentation and reflection on learnings. Since the early days of RUDi MRP work in Dawson Creek, the requests for virtual first-call coverage have continued to pour into the RTVS program, providing opportunities to implement these learnings.

### Strengths and Limitations

A strength of this study was its embedded approach as part of ongoing quality improvement, including the timeliness of data collection to capture experiences as closely to the time of occurrence as possible. Furthermore, the emphasis on understanding multiple perspectives through interviews both mirrors and facilitates perspective taking, a key ingredient or competency of collaborative practice [31,32]. It is hoped that documentation will provide a basis for adopting those things that resonate and adapting those that differ. While generalizability is not the objective of looking at 1 case, the limited nature of data collection and focus on 1 site limits its generalizability. Nevertheless, we hope our study will provide some insight and guidance for communities that follow and the cumulative evaluation as more community EDs are documented will provide a more substantial basis for extrapolation.

A major limitation is that there was no systematic collection of outcome data. Incorporating comparators in evaluation would further efforts to determine the safety of the strategy in terms of patient outcomes. While it would be challenging to conduct a randomized controlled trial to determine the effectiveness of this type of digital health strategy given its use in crises rather than as part of an ongoing service delivery strategy, incorporating population data comparators to test hypotheses related to quality, safety, economics, and other evaluation questions would strengthen the evidence. As an early case study focusing on provider experiences neither patients nor their family caregivers were interviewed to directly share their feedback and experiences, which would have greatly benefited quality improvement efforts. In our current RTVS evaluation activities, we have obtained an ethics review to conduct patient and family caregiver interviews to get first-hand feedback across patient-facing RTVS services, including this type of ED coverage. The ongoing albeit intermittent use of this virtual ED strategy in British Columbia offers opportunities for deeper quality improvement as well as further evaluation research. RTVS evaluation has deepened and become more programmatic since the time of this experience in Dawson Creek to address health outcomes; economic and equity-focused outcomes; and wider and deeper investigation of experiences of patients, families, providers, and stakeholders.

### Comparison With Prior Work

The primary success of the RUDi coverage was that the DCDH ED avoided going on diversion and that patients could continue to access urgent care locally. Reducing the need for transfer is a benefit of remote management of patients in EDs [13,15], which may reduce costs for the system and has both cost-related and other benefits for patients and families who are able to stay in the community [33]. RUDi physicians were able to provide or facilitate the necessary care for all patient cases during the period the strategy was in place, with a small number being transported out-of-community for additional medical care, which is similar to DCDH’s normal operations.

Leaders, clinical staff, administrators, and technical staff across stakeholder organizations were able to codevelop and implement this model of practice very rapidly in a crisis to prevent diversion. This involved planning meetings via videoconference that went late into the night, interprofessional and interagency collaboration, and creative problem-solving. Our findings emphasized that relationships and leadership were foundational to the success of the effort. Leaders and team members involved in the development and implementation shared a vision and took a flexible approach grounded in the needs of the community—factors relating to sustainability [14]. The organizations not only had a history of working together in partnership, but the people had strong relationships on a human level. The leaders participated as part of the team, were willing to take risks, and demonstrated the ability to understand different perspectives [31,32]. Digital health strategies such as these by their very nature crosses boundaries, the least of which may be geography. The unique contribution of this publicly funded virtual ED strategy is that it builds the capacity of teams in communities as it helps the system meet crises. While the benefits of RTVS in both rural and ED contexts resonate with our study, the RTVS safety net strengthens local teams who retain the skills acquired through remote collaboration and thus may contribute to the retention of local providers. It is a story of community development as much as a story of health service delivery in a crisis.

The virtual ED strategy provided a safety net for local patients and physicians challenged by the ever-present threat of burnout and achieved its goal of preventing diversion. DCDH medical staff noted that the digital coverage highlighted ongoing issues of physician recruitment and retention and the need for long-term solutions. This strategy has great potential to ease the pressures of an ongoing physician shortage, which has been amplified by the COVID-19 pandemic. As Mazurik et al [34] (p10) point out, “when a common goal must be urgently met, emergency care systems can rapidly adapt.” However, the DCDH remote model of practice not only aimed to provide overnight relief to short-staffed rural physicians, but the new clinical pathway also aimed to support nurses on the ground. While benefits for nurses were documented, their workflow changed the most substantially and required additional training and adjustments that raised questions surrounding the scope of practice across remote collaboration. Steps must be taken to ensure that digital health strategies do not address recruitment and retention challenges for physicians while worsening challenges for nursing staff, essentially trading physician burnout for nurse burnout [15].

Another issue related to sustainability was the need for extensive IT support both in starting up and implementing the strategy. Individuals involved “stepped up” and worked “above and beyond” for a shared goal. This included such acts as driving for 2 hours after the end of the workday to hook up equipment and navigating bureaucracy to obtain permissions for virtual physicians to access local systems. While a crisis necessitated this extraordinary effort, it is not sustainable for volunteerism to bolster efficiency. Funding and personnel for IT need to be in place to support the people within the virtual strategy.

Trust and relationships were foundational to successful collaboration resonates with organizational theory [35]. The collaborative development and implementation of this coverage was in itself a vehicle to strengthen relationships and build trust within the team and organization, which in turn may bolster continuity, recruitment and retention, and community capacity. Just as care is kept closer to home with the remote staffing, so too is the capacity and team of the local ED strengthened through the involvement of RTVS.

### Conclusions

The Dawson Creek experience showed that having a highly trained team of virtual physicians partnering with local health professional teams in times of staffing crisis can be done safely and effectively and should be looked at as an acceptable alternative to diversion. This case study provides insight into the process of developing and implementing this RTVS diversion prevention strategy. This case study, completed in the context of quality improvement, provides learnings to be implemented to serve future rural, remote, and Indigenous communities in crisis. Our study suggests that rapid codevelopment and implementation of a digital health solution is possible through leveraging partnerships and mutual trust between virtual and community providers. By establishing new and modified clinical workflows, RTVS was able to provide a safety net for rural patients and providers challenged by burnout.

## Abbreviations

CTAS: Canadian Triage and Acuity Scale
DCDH: Dawson Creek District Hospital
ED: emergency department
MRP: most responsible provider
NHA: Northern Health Authority
RTVS: real-time virtual support
RUDi: Rural Urgent Doctor in-aid

## Data Availability

The data sets generated and analyzed during this study are not publicly available due to being generated and analyzed as part of an internal quality improvement initiative but are available from the corresponding author on reasonable request.
